# Experiences Related to Patients and Families’ Expression of Spiritual Needs or Spiritual Support Within Healthcare Settings During the COVID-19 Pandemic: A Scoping Review

**DOI:** 10.1007/s10943-022-01556-y

**Published:** 2022-04-19

**Authors:** Michael Connolly, Fiona Timmins

**Affiliations:** 1 School of Nursing, Midwifery and Health Systems, University College Dublin, Dublin, Ireland; 2 Our Lady’s Hospice and Care Services, Dublin, Ireland

**Keywords:** Spirituality, Spiritual needs, Spiritual supports, COVID-19, Experiences healthcare chaplains

## Abstract

The aim of this review was to explore the evidence surrounding patients and families’ expression of spirituality, spiritual needs or spiritual support within healthcare settings during the COVID-19 pandemic from the perspective of nursing practice. While there is a plethora of research and publications related to COVID-19 and there are reports of increasing attention to nurses’ psychological distress, there is little understanding of experiences related to patients’ expression of spirituality, spiritual needs or spiritual support within healthcare settings during the COVID-19 pandemic. A scoping review was conducted to search and select potential studies and undertake data extraction and synthesis. Twenty-one studies published between March 2020 and August 2021 were identified. Themes and subthemes that emerged from analysis of the studies included spiritual needs, new awareness of spiritual needs and spiritual interventions, chaplaincy referrals, and improved well-being. The potential requirement for spiritual care during these times has anecdotally never been greater. At the same time the existent ethical challenges persist, and nurses remain reticent about the topic of spirituality. This is evident from the clear lack of attention to this domain within the published nursing literature and a limited focus on spiritual care interventions or the experiences and spiritual needs of patients and their families. Greater attention is needed internationally to improve nurses’ competence to provide spiritual care and to develop and advance nursing and research practice in the field of spiritual care.

## Introduction

Spiritual support within healthcare has had a long and distinguished history (Swift, 2014). Steeped within religious traditions, western healthcare has been systematically uncoupling from these traditions in keeping with growing secularism within the context of modern societies (Nissen et al., 2021). However, healthcare chaplaincy and pastoral care services, which arose from this legacy, remain intrinsic to the provision of healthcare across societies at large, for the simple reason that people require this support.

Religious organisations, often the founders of nursing programmes or philanthropic providers of healthcare services, have less influence in today’s modern healthcare environment. Healthcare chaplaincy and pastoral care services are emerging as a professional service, increasingly multifaith (Brady et al., 2021), that support patients, families and staff (Tata et al., 2021). While there are some legacy issues that continue to raise concerns, in some jurisdictions, including the presence of Christian iconography and chapels within healthcare settings and indeed challenges to the need for chaplaincy services (Swift, 2014, *TheJournal.ie*, 2013; Medical Independent, 2013; National Secular Society, 2009), the benefits of or requirements for spiritual support in healthcare are reported by people of all faiths, and none, and there is increasing attention paid to the spiritual aspects of care even within highly secular countries (La Cour & Hidvt, 2010).

End-of-life care decisions (Clyne et al., 2019; Tata et al., 2021), for example, and attitudes to death and dying (Thauvoye et al., 2020) are often profoundly embedded in personal and cultural beliefs, and addressing spirituality can serve to support end of life in a positive way (deVries et al., 2019). The healthcare chaplain and pastoral care workers therefore have a key role in spiritual support in healthcare across the spectrum of illness experiences (Nuzum, 2016; Nuzum et al., 2021), and where this facility is available, they are the specialists in spiritual support for patients and their families.

At the same time, there is a growing interest in spiritual care provision by nurses across the globe (Fang et al., 2022; van Leeuwen et al., 2021; Wu et al., 2016). Indeed, there are moves internationally for spiritual care and spiritual support to form part of the nurse’s role, most recently elucidated within the European Erasmus Plus Project- *Enhancing Nurses and Midwives' Competence in Providing Spiritual Care through Innovation, Education and Compassionate Care* (EPICC, 2021). The latter provides clear guidance for nurses to support patients’ spirituality, through the identification of four distinct nursing competencies: (i) intrapersonal, interpersonal and spiritual care assessment, (ii) planning, (iii) spiritual care intervention and (iv) evaluation perspectives. These competencies support the nurse’s awareness of their own spirituality in order to be able to comprehensively assess spiritual care needs and provide spiritual care interventions (EPICC, 2021). These activities are carried out in close collaboration with healthcare chaplains, where relevant, as referral to chaplains or pastoral care services are a key feature within these competencies. For nurses, EPICC define spirituality as:

“The dynamic dimension of human life that relates to the way persons (individual and community) experience, express and/or seek meaning, purpose and transcendence, and the way they connect to the moment, to self, to others, to nature, to the significant and/or the sacred” (EPICC, 2021)

While there is growing interest in the nurse’s role in the provision of spiritual support (van Leeuwen et al., 2021), one would expect this to have been reflected and indeed exacerbated in the literature during the COVID-19 pandemic. While new ways of providing end-of-life care and other types of care certainly emerged (Bowers et al., 2021), and from the public’s perspective spirituality seemed to have become increasingly valued  (Papadopoulos et al., 2020), it is important to know whether or not supporting spiritual needs by nurses increased within this context and what support patients and families required.

Given the perceived importance of spiritual care, this area would likely receive some attention during a crisis point in healthcare and within individual’s lives. To address this gap, we performed a scoping review to explore the spiritual support of patients and families during COVID-19 from the perspective of nurses.

## Aim of the Review

The aim of this scoping review was to examine the literature exploring healthcare patients’ and families’ experiences of spirituality, spiritual needs or spiritual support within healthcare settings during the COVID-19 pandemic. Spirituality is understood as a dimension of personal life that enables the expression and seeking of meaning, the value of connectedness and for some, transcendence (EPICC, 2021; Weathers et al., 2016).

## Methods

A scoping review was conducted in order to explore the nature of current evidence (Armstrong, 2011; Peters et al., 2020). The review primarily focussed on literature from March 2020 to March 2021, reflecting the early outset of the COVID-19 pandemic (although no exclusionary dates were applied to the search).

### Identifying the Relevant Studies

The search strategy was based on clear search terms listed in Table 1 and further refined by applying inclusion and exclusion criteria (Table 2). Database searches were undertaken in CINAHL, Medline, Atla Religion Database with Atla Serials Search terms.

**Table 1:** Search terms

Concept 1: Spirituality
(MH &quot;Spiritual Well-Being (Iowa NOC)&quot;) OR (MH &quot;Spiritual Support (Iowa NIC)&quot;) OR (MH &quot;Spiritual Distress (NANDA)&quot;) OR (MH &quot;Spirituality&quot;) OR (MH &quot;Psychological Well-Being&quot;) OR &quot;spiritual&quot; OR (MH &quot;Spiritual Distress (Saba CCC)&quot;) OR (MH &quot;Spiritual Comfort (Saba CCC)&quot;) OR (MH &quot;Potential for Enhanced Spiritual Well Being (NANDA)&quot;)
Concept 2: COVID
COVID-19 or Coronavirus

**Table 2:** Inclusion and exclusion criteria

Inclusion criteria	Exclusion criteria
All papers that explore spirituality from patients’ and families’ perspectives	All papers that explore spirituality from healthcare workers’ [or others’] perspectives without reference to healthcare recipients [e.g. patients’ and families’] experiences or needs [e.g. attitudinal measures]
All papers that provide an outline of spirituality or spiritual care interventions or guidance for healthcare recipients [e.g. patients’ and families’]	All papers that discuss healthcare workers’ roles in relation to spirituality [chaplaincy for example] without reference to healthcare recipients [e.g. patients’ and families’] experiences or needs
All papers that provide a discussion on spirituality or spiritual care interventions or guidance for healthcare recipients [e.g. patients’ and families’]	Papers that discuss related subelements of spirituality [such as connectedness] with no reference to spirituality
Papers that satisfy the inclusion criteria ought to include reference to the term spirit, spiritual or spirituality as a basic	

### Study Selection

**Fig. 1:**
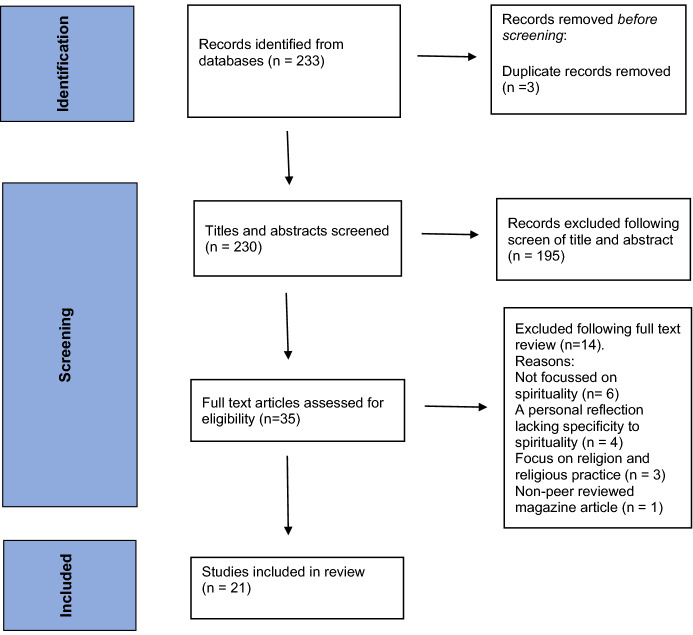
PRISMA statement (Page et al., 2021b)

Both researchers screened all titles and abstracts and undertook full-text review. The results are reported using a PRISMA flow chart (Page et al., 2021a) (Fig. 1).

### Charting the Data

One researcher undertook preliminary data extractions using a standardised form to chart the data. This was later confirmed by the second researcher. Study design, population, sample size, setting, conduct, findings and reported limitations were considered. Information on interventions was extracted where appropriate. Both researchers reflected on the quality of the studies included. Quality appraisal using specified criteria was not undertaken as this was a scoping rather than a systematic review (Munn et al., 2018). Thematic analysis was undertaken using a three-step approach of firstly coding the material and then identifying themes and finally constructing a thematic network (Dhollande et al., 2021). Initial coding provided the opportunity to highlight text, into phrases and sentences. Coding provides a condensed version of the main points and common meanings that occur in the data (Thomas & Harden, 2008). The codes created were then reviewed and patterns identified which when taken together enable the generation of themes. The constructed thematic networks summarise the main themes and are linked back to the aim of the review (Schellekens et al., 2020).

## Findings

### Selection of Studies

The search yielded 233 records (Fig. 1). Three duplicates were removed. Following title and abstract review 195 records were excluded. Key reasons for exclusions were lack of relevance and incompatibility with the study aims and scope. Thirty-five studies underwent full full-text review, 14 were excluded, mainly because these did not fully meet the inclusion criteria. Twenty-one studies were ultimately included in the review.

### Description of the Studies

**Table 3:** Characteristics of the studies

Author (year)	Country	Setting	Information about the intervention	Type of study	Method	Sample	Findings
Bakar et al. (2020)	USA	City based palliative care team	No intervention	Not indicated	Reflection on provision of palliative care during COVID-19 by a palliative care team	N/A	Spirituality has become increasingly important to families during COVID-19 pandemic
							Chaplains play a vital role,
							Chaplains have facilitated at the request of families to play recordings of prayers on devices for patients with COVID-19
Damani et al. (2020)	India	N/A	No intervention	Position Statement	Position Statement	N/A	Palliative care needs to be adapted and included in mainstream medical care to offer compassionate care appropriate to the need within the limitations of time, isolation, and resource availability
De Diego-cordero et al. (2021)	Spain	Critical care and emergency departments	No intervention	Qualitative	In-depth interviews	19 ICU nurses	During the pandemic nurses provided spiritual care to their patients
							Although nurses believe spiritual care is important to help patients the lack of an agreed definition of spirituality was noted
							Workload and lack of sufficient time and training were seen as barriers to providing spiritual care
Dein et al. (2020)	UK	N/A	No intervention	Editorial	Editorial	N/A	Limited research on implications of COVID-19 for religion and mental health
Drummond and Carey (2020)	Australia	Older care centre	Identified use of WHO Spiritual Care Intervention codings to explore provision of spiritual care	Qualitative	Case Study focussed on care centre and consideration of practitioners experience	One care centre	Isolation protocols during COVID-19 have triggered further needs for residents
							Impact on staff
							Impact on families
							Lack of closure around death
Finiki and Maclean (2020)	Aotearoa New Zealand	Hospital	No intervention	Not indicated	Personal reflection on authors experience	Two spiritual pastoral therapists	Providing spiritual care services to nurture well-being is challenging
							Spirituality has been integrated into all areas of hospital life
Galehdar et al. (2020)	Iran	Covid-19 wards of general hospitals	No intervention	Qualitative	Conventional content analysis	20 nurses caring for patients with Covid-19	Fear of death was reported to be stressful and annoying for patients
							Patients were concerned about how they were perceived by society
							Patients need to receive high-quality health services
							The provision of spiritual care can reduce stress and improve feelings of wellness
Geppert and Pies (2020)	USA	N/A	No intervention	Not indicated	Personal reflection on authors experience	N/A	COVID-19 has enhanced spirituality and religion for many Americans
							Downside is that faith can be used to give credence to vaccine hesitancy—‘Jesus is my vaccine.’
							Psychiatry can play a useful role in re-framing distress aspects of COVID-19 pandemic
Giffen and MacDonald (2020)	Scotland	Chaplaincy services in primary care	No intervention	Quantitative	Survey questionnaire	13 chaplains	COVID-19 is a defining moment in lives of patients and their biopsychosocial spiritual needs
							Due to complex presentations equitable access to chaplaincy needs to be assured
Gray-Miceli et al. (2020)	USA	N/A	No intervention	Qualitative	Case Vignette including author opinion	N/A	Socialisation in assisted living environments allows for integration of body, mind and spirit
							Nurses’ aides have and continue to have a key role in providing for the social needs of older adults in assisted living centres
Hashmi et al. (2020)	Pakistan	N/A	No intervention	Not indicated	Personal reflection on authors experience	N/A	Inclusion and collaboration of spiritual leaders with healthcare professionals is needed to ensure holistic care and avoid religious stigma
Heidari et al. (2020)	Iran	N/A	No intervention	Letter to the Editor	Letter to the editor	N/A	Patients feel the need for spiritual car
							Developing a stronger relationship with ‘God the Almighty’ leads to reduced stress and anxiety and increases hope and calmness
							To provide holistic care spirituality must be included in the design of health systems
Pierce et al. (2021)	USA	Emergency Department	No intervention	Short Report	Reflection based on authors experience	N/A	When a death has occurred in the ED where the spiritual or religious preference is known, a member of the team who has had training should lead a time for reflection following the death
							The use of non-pastoral spiritual care teams, who have been trained by chaplains should be used to lead such reflective interventions
							Tele-medicine should be used to support communication between chaplain and patient and chaplain and family
Pies (2020)	USA	N/A	No intervention	Not indicated	Personal reflection on authors experience	N/A	Each person needs to find the unique path that leads from grief to healing
							Those who do not embrace religious or spiritual practices may find solace in the therapeutic use of music, poetry or literature
Rathore et al. (2020)	India	N/A	No intervention	No indicated	Personal reflection on authors experience	N/A	Applying CARE is useful in COVID-19 pandemic
							Emotional well-being is essential for physical well-being
							Spirituality care involves the care of the patient as whole
Rao et al. (2020)	India	N/A	No intervention	Review	Quick review	N/A	Palliative care needs to address the rapidly changing situation caused by COVID-19
							The sense of connectedness that is part of spirituality is threatened in a tie of a pandemic
							Spiritual care helps to promote adaptation and foster resilience through overcoming fear and finding hope and meaning in a time of uncertainty
Rentala and Ng (2021)	India	Subject’s own home	Integrated Body-Mind-Spirit (IBMS) Intervention	Qualitative	Case Study focussed on one patient	Single subject	Improved well-being of COVID-19 patient when IBMS (integrative mind–body-spirit) was implemented
							IBMS can be implemented through mobile phones/videos
Roman et al. (2020)	South Africa	N/A	No intervention	Not indicated	Personal reflection on authors experience	N/A	Provision of spiritual care contributes to improved patient well-being
							Spiritual care is regarded as life-enhancing and a coping resource
Sanchetee and Sanchetee (2020)	India	N/A	No intervention	Not indicated	Personal reflection on authors experience	N/A	Looking after our own needs is important during a pandemic
							Others in society should not be put in danger or inconvenienced during a pandemic
							Personal philosophy and religious belief should be reinterpreted in the present context
Umucu and Lee (2020)	USA	N/A	No intervention	Quantitative	Cross-sectional survey design Survey questionnaire	269 individuals with self-reported disabilities and chronic illnesses	Acceptance and self-distraction were most common coping strategies
							Perceived stress was associated with maladaptive and adaptive behaviours
							Active coping, denial, use of emotional support, humour, religion, and self-blame were associated with participants well-being
Wiederhold (2020)	Unknown	N/A	No intervention	Editorial	Editorial	N/A	A reason for persistence of religious and spiritual practice is that both are designed to help during difficult times
							Physical and mental health benefits to religion and spirituality can be found across traditions and cultural divides
							Dedication and flexibility are two qualities of religious and spiritual communities

Of the 21 studies (Table 3), five were qualitative, with three using case study method (Drummond & Carey, 2020; Gray-Miceli et al., 2020; Rentala & Ng, 2021) and two used content analysis of interview data from nurses (de Diego-Cordero 2021; Galehdar et al., 2020). Two studies were quantitative (Giffen & MacDonald, 2020; Umucu & Lee, 2020). Nine studies were commentaries based on personal reflections of experiences during COVID-19, (Bakar et al., 2020; Finiki & Maclean, 2020; Geppert & Pies, 2020; Hashmi et al., 2020; Pierce et al., 2021; Pies, 2020; Rathore et al., 2020; Roman et al., 2020Sanchetee & Sanchetee, 2020). Of the remaining five studies, three were either editorials or letters to the editor (Dein et al., 2020; Heidari et al., 2020; Wiederhold 2020), one was reported by the authors as a ‘quick review’ (Rao et al., 2020) and one study provided a position statement (Damani et al., 2020). None of the studies involved interventions.

#### Generation of Themes

Following an analysis of the extracted data, two main themes and five subthemes emerged from analysis of the studies: spiritual needs, including additional spiritual needs in the context of COVID-19 and new awareness of spiritual needs; and spiritual interventions, including some novel interventions, chaplaincy referral and improved well-being.

### Spiritual Needs

All of the studies demonstrated awareness of the significance of spirituality in healthcare. A number of the studies highlighted the increased reported significance of spiritual needs during the COVID-19 pandemic. In particular, the significant role that healthcare chaplains and nurses played in providing spiritual care, in the form of presence and prayer and ensuring that meaningful spiritual objects were provided to patients as needed (Bakar et al., 2020; de Diego-Cordero et al., 2021; Geppert & Pies, 2020; Rathore et al., 2020). It is important to note that three of these papers (Bakar et al., 2020; Hashmi et al., 2020; Pierce et al., 2021) are based on both personal experience of providing frontline care during the COVID-19 pandemic.

Other studies demonstrated a new awareness of spiritual needs particularly those associated with COVID-19 pandemic (Drummond & Carey, 2020; Gray-Miceli et al., 2020; Hashmi et al., 2020). This new awareness was highlighted by de Diego-Cordero et al. (2021) study of nurses working in the critical care setting, where nurses saw the value of spiritual care in helping patients deal with their diagnosis of COVID-19 but viewed their limited education on the topic and lack of time as barriers to providing this care. These findings were also highlighted by Drummond and Carey (2020) and Gray-Miceli et al. (2020) studies, both conducted in older care centres, where the lessening of socialisation and the impact of isolation on patients/residents, staff and families during COVID-19 was explored.

In an opinion piece, Hashmi et al. (2020) suggest the need to recognise the collaborative role that spiritual care providers, such as healthcare chaplains and pastoral care workers have in ensuring that religious bias is avoided and spiritual care is embedded in the holistic care provided to patients, particularly in a time of the COVID-19 pandemic.

### Spiritual Interventions

Spiritual care is considered within the context of holistic nursing care. A number of the included studies make reference to spiritual interventions, and in some cases, novel interventions to support spiritual care are identified (Pierce et al., 2021; Pies, 2020; Rathore et al., 2020; Rentala & Ng, 2021). Pierce et al. (2021) spoke from their experience of providing spiritual care in the emergency department. They highlighted a new approach to providing spiritual care through the use of an algorithm for spiritual care provision in the event of termination of resuscitation during the pandemic.

Pies (2020) in his opinion piece, suggests that for some who do not embrace spiritual or religious practice, solace may be found in the therapeutic use of music, poetry or literature. Based on findings from their case study, Rentala and Ng (2021) suggest the use of integrative mind–body-spirit approach as a novel way to promote spiritual well-being and maybe helpful in providing insight to nurses working in mental health services of the importance of including psycho-social-spiritual interventions. Recognising the relationship of body-mind-spirit has the potential to facilitate a more holistic approach to care that promotes not just physical but also spiritual well-being (Rentala & Ng, 2021).

A number of the studies included in this review indicated the effect that spiritual care had on increased hope, resilience and well-being (de Diego-Cordero et al., 2021; Finiki & Maclean, 2020; Galehdar et al., 2020; Rao et al., 2020; Roma et al., 2020; Umucu et al., 2020; Wiederhold, 2020). While this effect is for the most part based on personal opinions of the authors, Galerhar et al. (2020) and Umucu et al. (2020) based their findings from studies involving nurses and patients, respectively. Galehdar et al. (2020) indicated that provision of spiritual care had a positive impact by reducing stress and improving feelings of wellness in patients cared for by nurses in Iran, while Umucu et al. (2020) found that spiritual care increased hope and resilience and ultimately improved well-being.

Finiki and Maclean (2020), Rao et al. (2020), Roma et al. (2020), and Wiederhold (2020) in their opinion pieces all supported this view. Giffen and MacDonald (2020) in reporting of the findings on spiritual care during the COVID-19 pandemic indicated that access to healthcare chaplaincy was important due to the significant role that they played supporting patients and families during the various stages of the COVID-19 pandemic (Giffen & MacDonald, 2020).

## Discussion

Spirituality is understood as fundamental aspect of humanity that relates to belonging, finding personal meaning, peace and a sense of connection to others (Coppola et al., 2021). While spirituality and fulfilling spiritual needs are not necessarily associated with religion, some will express their spirituality in this way (Coppola et al., 2021; Weathers et al., 2016). This review found that while spiritual needs were perceived as important, is it notable that much of the literature is from the perspective of the nurse, or healthcare chaplain, rather than the patient or family, and much of this is anecdotal. However the provision of spiritual objects remained important for some (Bakar et al., 2020; de Diego-Cordero et al., 2021; Geppert & Pies, 2020; Rathore et al., 2020), although caution was advised in terms of ensuring that spiritual care remains sensitive to faith and non-faith requirements (Hashmi et al., 2020).

This support through the provision of spiritual objects was interesting, as spirituality and religiosity are often shaped and influenced by culture (Murgia et al., 2020), and individuals’ experiences (Nascimento et al., 2016). Providing person centred spiritual care can be an important dimension of dignified care in multicultural contexts (Cheraghi et al., 2014). Indeed, it is found that people find solace in rituals, conversations, and attention, provided by pastoral care workers and healthcare chaplains (Brady et al., 2021). Indeed, Imber-Black (2020) has recently identified how families and communities preserved and developed religious and other rituals to connect during COVID-19.

Certainly spiritual support with or without belief in God can give people a sense of meaning, and help with coping, especially at the end of life (Clyne et al., 2019, Nuzum et al., 2021), and nurses have been encouraged to provide spiritual support (Clarke, 2013). However, addressing spirituality within healthcare settings is not a straightforward issue, given the complexities and diverse spiritual (and non-spiritual) beliefs and practices that exist. While evidence from research indicates that spirituality has the potential to support optimal health (Peteet et al., 2018), since it is strongly associated with well-being (Forlenza & Vallada, 2018), higher quality of life and psychosocial experience (Koenig et al., 2012; Labrague et al., 2016), this review found that nurses still lack education and experience in this area and find it difficult to prioritise time for this activity (de Diego-Cordero et al., 2021). Furthermore, there was very little evidence based research that explored the effects of spiritual support.

In their work, da Silva et al. (2019) demonstrated the benefits of spiritual support for those coping with breast cancer. However, nurses are not always aware of such benefits as the development of nurses’ competencies is a very recent initiative (EPICC, 2021; van Leeuwen et al., 2021), and there appears to be a lack of nursing research in this area. While the review found that nurses had a commitment to providing spiritual support and believed that patients and families found this important, much is needed to strengthen and support nurses’ roles and understandings around spiritual care provision, and to drive applied research in this field.

 It is unclear from this review how spirituality is expressed from either the patient or family perspectives. It was also unclear, given the dearth of literature, what the nature of the issues were as experienced and encountered by individuals and families with regard to spirituality within healthcare in the context of a pandemic. The complexity and diverse nature of patients’ and families’ needs, clearly warrants attention. There are a range of spiritual needs (physical, emotional, cognitive, psychosocial, behavioural) and felt needs limitedly expressed by patients and families; however, this review revealed an account that was biased toward the perspectives of healthcare chaplaincy and nurses. Furthermore, the very far reaching role that healthcare chaplains (Timmins et al., 2018) and indeed nurses can play (EPICC, 2021) in providing spiritual support was under reported as the studies dealt merely with interventions such as presence, prayer and the provision of meaningful spiritual objects.

Key aspects of expressed needs within context of studies were not addressed in full such as the fear of death, lack of closure around death spiritual distress or other existential associated concerns (Drummond & Carey, 2020; Galehdar et al., 2020). However, this finding is not surprising, and a recent review that explored media coverage of spiritual support during COVID-19 (Papadopoulos et al., 2020) reflected a similar lack of specifics in relation to care provision during this time. Furthermore, these authors noted that while spiritual support was highly valued, there was “inadequate beside spiritual support” (Papadopoulos et al., 2020:104). They also highlighted gaps in staff education and training in this topic and the need for a “national spiritual support strategy for major health emergencies and disasters” (Papadopoulos et al., 2020).

However, one positive and important step in improving nurses’ understanding of spirituality and spiritual care provision is the recent initiation of the Erasmus Plus Project “From Cure to Care, Digital Education and Spiritual Assistance in Healthcare” (2021, Timmins et al., 2022). This project aims to develop educational resources for nurses to provide spiritual care. An E-Learning programme to support religious-spiritual competencies within a multicultural perspective will be developed that hopes to address’s national and international gaps in nurses’ knowledge and skills and improve their confidence in support patients’ spiritual needs.

It is hoped that this emergent body of knowledge, competencies and specific tools related to spiritual care provision begins to provide the guidance and support that urgently needed across healthcare settings internationally. Hopefully, this European initiative, along with ongoing work by EPICC (2021) in the field, will also spearhead the much needed research in the area of nursing and spirituality, which is urgently needed not only to implement and evaluate nurses’ competencies but to determine the effect of these and best practice in relation to spiritual care interventions by nurses.

## Conclusion

While there is a plethora of research and publication related to COVID-19 and reports of increasing attention to nurses’ psychological and moral distress (Hossain & Clatty, 2021), there is little understanding of experiences related to patients’ or families’ expression of spirituality, spiritual needs or requirement for spiritual support within healthcare settings during the COVID-19 pandemic from the perspective of nurses. This scoping review revealed very little empirical material related to this topic.

The lack of attention to spirituality and spiritual care by nurses is not surprising, because although there are national and international requirements regarding the provision of spiritual care, recent reviews have found that spiritual care is largely omitted from practice (Hvidt et al. 2020; Whelan, 2019). At the same time, healthcare chaplains working in the frontline of healthcare anecdotally report an increased and intensive demand on the services (Busfield, 2020), and the various responses and challenges for healthcare chaplaincy internationally, including adapting to the use of technology to provide pastoral care (Byrne & Nuzum, 2020; Carey et al., 2020).

COVID-19 has had far reaching consequences on the healthcare system. Considerable attention has been paid within the literature to the effect of the pandemic on healthcare staff, with less attention on the effects on patients and families. Certainly this occurrence has resulted in stress for all parties; however, this is likely magnified for patients and families who find themselves at times of health crisis or witnessing end of life. The potential contribution of COVID-19 to illness and the restrictions imposed by the distancing required, served to render what are already challenging situations, to uniquely stressful ones.

The potential requirement for spiritual care during these times was anecdotally greater than ever, yet at the same time challenges remain, and nurses remain reticent about the topic, evidenced by the clear lack of attention to this domain within the published literature. More needs to be done internationally to imbed newly developed standards for nurses (EPICC, 2021) into healthcare practice and to develop and advance nursing and research practice in the field of spiritual care, and to continue to explore and develop innovate ways to support an increase in knowledge, skills and competencies among nurses globally (Timmins et al., 2022).

## Limitations

One limitation of this review is that the search terms do not potentially capture the breadth of the literature in this area globally. The time period is also restricted to the advent of COVID-19, and therefore, this provides only a particular time sensitive view of the literature.
